# Tricho-Dento-Osseous Syndrome: Diagnosis and Dental Management

**DOI:** 10.1155/2012/514692

**Published:** 2012-08-27

**Authors:** Ola B. Al-Batayneh

**Affiliations:** Department of Preventive Dentistry, Faculty of Dentistry, Jordan University of Science and Technology, P.O. Box 3030, Irbid 22110, Jordan

## Abstract

Tricho-dento-osseous (TDO) syndrome is a rare, autosomal dominant disorder principally characterised by curly hair at infancy, severe enamel hypomineralization and hypoplasia and taurodontism of teeth, sclerotic bone, and other defects. Diagnostic criteria are based on the generalized enamel defects, severe taurodontism especially of the mandibular first permanent molars, an autosomal dominant mode of inheritance, and at least one of the other features (i.e., nail defects, bone sclerosis, and curly, kinky or wavy hair present at a young age that may straighten out later). Confusion with amelogenesis imperfecta is common; however, taurodontism is not a constant feature of any of the types of amelogenesis imperfecta. Management of TDO requires a team approach, proper documentation, and a long-term treatment and follow-up plan. The aim of treatment is to prevent problems such as sensitivity, caries, dental abscesses, and loss of occlusal vertical dimension through attrition of hypoplastic tooth structure. Another aim is to restore function of the dentition and enhance the esthetics and self-esteem of the patient. This paper proposes treatment approaches that include preventive, restorative, endodontic, prosthetic, and surgical options to management. In addition, it sheds light on the difficulties faced during dental treatment of such cases.

## 1. Introduction

Tricho-dento-osseous (TDO) syndrome is a rare, autosomal dominant disorder first distinguished by Lichtenstein et al., in 1972 [1], and is characterised by hair, dental, bone, and other defects. Most of the knowledge about TDO is from case reports and genetic studies in the literature, the first case was reported in 1966 by Robinson and coworkers [2–11]. 

The importance of TDO to dentists is for two reasons; the severe hypoplastic defects of enamel and the difficulty in differentiation from hypomaturation-hypoplastic amelogenesis imperfecta with taurodontism from clinical and radiographic criteria alone, which necessitates further histologic and genetic investigations. The clinical features of TDO concerning bone, hair, and nails are variably phenotypically expressed and sometimes may be missing which make the diagnosis somewhat confusing.

The purpose of this paper of TDO is to explore the diagnostic criteria, the main clinical and radiographic findings that distinguish TDO from other conditions confused with it in the literature, and to suggest a comprehensive management focusing on dental treatment approaches for patients with TDO. 

## 2. Mode of Inheritance 

All previously published cases have shown that TDO is most likely transmitted in a highly penetrant autosomal dominant manner [1–9, 12]. Genetic analysis in most studies has shown a mutation in the DLX3 gene [11, 13, 14]. Genetic linkage was identified on chromosome 17q21 [15] and a 4 base-pair (4 bp) deletion in the DLX3 gene has also been identified. The 4 bp deletion causes a frameshift mutation and formation of a termination codon; this leads to a truncated protein able to potentially bind to DNA, but due to the intact homeodomain region, it is functionally altered. This was the first human study to show the importance of the DLX gene family in the development of hair, teeth, and bones [13].  

It is not known whether the variable clinical features in TDO are the result of genetic heterogeneity or clinical variability. Price et al. [10] suggested that the variable clinical phenotype observed in families from Virginia and North Carolina indicates that clinical variability was not the result of genetic heterogeneity at the major locus but may reflect genetic heterogeneity at other epigenetic loci and/or contributing environmental factors. For diagnostic purposes, genetic testing aims to test the DNA of suspected patients for the presence of a DLX3 deletion using PCR amplification [13]. During early development DLX3 is expressed in the placenta and plays a crucial role during embryonic development. It is also expressed in the first and second branchial arches and their derivatives including craniofacial bone. Later during development, DLX3 is expressed in structures involving epithelial-mesenchymal interaction such as teeth, hair follicles, and skin [16].

Choi et al. [17] verified that the DLX3 deletion mutation occurring in TDO increases osteogenesis by encouraging mesenchymal cell differentiation to an osteoblastic lineage and hastens the differentiation of osteoprogenitor cells to osteoblasts during later stages of osteogenesis. The authors also showed that cells overexpressing DLX3 in TDO demonstrate an increase in alkaline phosphatase activity, mineral deposition, and osteocalcin promoter activity, which is consistent with the increased bone density observed in TDO patients. On the other hand, Duverger and coworkers have shown that although the DLX3 deletion mutant in TDO is targeted to the nucleus it is not able to bind DNA, and therefore, its transcriptional activity is altered. However, when both DLX3 isoforms are coexpressed in heterozygous cases, the DLX3 deletion mutant can interact with the DLX3 wild-type isoform and form a complex that can indirectly bind DNA and exert a dominant negative effect on the DLX3 wild-type transcriptional activity. Therefore, where both isoforms are coexpressed, these findings could not account for the phenotype in TDO patients [18].

An *in vivo* study of DLX3 deletion mutant transgenic mice also suggested a novel role in osteoclast differentiation and bone resorption mediated by IFN-*γ*. Increased expression of IFN-*γ* by immune cells has been associated with decreased osteoclastic bone resorption, contributing to enhanced trabecular bone volume and mineral density in DLX3 deletion mutant transgenic mice. This suggested a novel role for this DLX3 mutation in osteoclast differentiation and bone resorption [19].

Interestingly, it has been recently found that the DLX3 deletion mutant in generated transgenic mice acts to disturb odontoblastic cytodifferentiation leading to apoptosis and aberrations of dentin tubule formation and dentin matrix secretion, resulting in a small amount of mature dentin and taurodontism. There was radiographic evidence of dentin defects in all teeth and the size of the pulp was enlarged. Findings from the study indicated that the DLX3 deletion mutant had different *in vivo* effects on odontoblasts compared to osteoblasts, where the amount of tooth dentin is decreased, while bone matrix apposition and mineralization increase since there was no evidence for apoptosis in alveolar bone cells in the transgenic mice. In summary, it seems that DLX3 deletion mutant has differential effects on matrix production and mineralization in dentin and bone [20]. Furthermore, a recent study identified two missense mutations in the DLX3 homeodomain upon mutational analysis of DLX3 in two affected Finnish families. The clinical features were described as markedly severe in one of the families in which the affected members developed only lanugo-type hair and severe dental anomalies. It was suggested that TDO is basically caused by loss of function and haploinsufficiency of DLX3 gene [21].

## 3. Characteristic Defects in TDO

A summary of the defects in TDO that have been mentioned in the literature are shown in Table 1, these include the following:

### 3.1. Hair Defects

Kinky or tightly curled hair at birth may be a characteristic and distinguishing feature in many families and aid in diagnosing TDO from hypomaturation-type amelogenesis imperfecta [1–3, 22–25]. Some families have been reported to have wavy hair [7, 24] or curly hair at birth that straightened out a few years later [6]. Seow [12] reported that the hair defects may vary among affected members of the same family.

### 3.2. Dental Defects

The dental defects in TDO are usually the most severe and reliable of all signs; they include yellow-brown discolored teeth [1–6, 9, 22–24]. The teeth display hypocalcification or hypomaturation enamel defects in association with enamel hypoplasia [1–8, 22–25]. The enamel is very thin (estimated to be 1/4 to 1/8 normal thickness) [12]. There is severe attrition of enamel [1, 2, 5, 8, 22, 23, 25], and this may be a cause for the commonly reported dental abscesses [1–4]. Seow [26] pointed out that taurodontism, a consistent feature in TDO, is always severe and involves all the molars, especially the mandibular first permanent molar. In fact, taurodontism in both the primary and permanent dentitions and enamel hypoplasia are fully penetrant features observed in all affected TDO individuals [13].

Histological sections of teeth in TDO show that the enamel is hypocalcified and decreased in thickness, [2, 3, 7] and the pulp chamber is enlarged with the pulp horns reaching the dentinoenamel junction, [2, 3, 6, 7], similar to what is seen in vitamin-D-resistant rickets [27]. Also, small amounts of interglobular dentin have been noted in a few teeth; [6] but Seow [12] indicated from a histologic study that there are no structural changes in the dentin. This fact is of interest because most of the syndromes involving bone defects are usually accompanied by changes in the dentin [12].

### 3.3. Bone Changes

While the hair and dental defects in affected patients with TDO usually occur at an early age, osseous changes happen to arise later throughout adulthood [15]. There is no consensus on the presence of sclerosis of cortical bone in TDO. It has been noted in some families with TDO [1–3], reported to be absent [2, 6] or not reported in other families [4, 5, 9, 22, 25]. Bone sclerosis may be a variable feature among members of an affected family; it is commonly reported in areas such as the base of the skull, the mastoids, and zones of provisional calcification in the long bones [28]. The concerns related to bone thickening are that it may lead to macrocephaly [7] and predispose to bone fractures [27]. However, Hart et al. [15] stated that this increase in bone thickness and density is not associated with any evident pathology.

### 3.4. Nail Defects

Nail defects include splitting of the superficial layers of the nails [1–3, 7, 8]. Sometimes, only some toenails may be affected [27]. Like the hair defects, there is also variability in the expression of nail defects among TDO patients [12].

### 3.5. Craniofacial Defects

These defects are somewhat inconsistent because of lack of enough cephalometric data for TDO patients. They include frontal bossing, [1, 7] square jaw, [1] mandibular prognathism, [2] and dolichocephaly [1, 9]. A recent cephalometric study reported maxillary retrusion [29].

### 3.6. Other Reported Abnormalities

Other abnormalities that have been reported in previous TDO cases include impacted teeth, [4, 7] clinodactyly, [1] and skin lesions [2].

## 4. Radiographic Features of TDO

Over half of the patients studied by Lichenstein and co-workers [1] whose dental abnormalities were further analyzed by Jorgenson and Warson [4] presented with single or multiple abscesses resulting from microexposures of the dental pulp following attrition or fracture of the enamel. Hart et al. [15] reported that osseous changes were variably expressed in a sample of patients with TDO and these included increased thickening of cortical bone (65%), obliterated diploe (68%), lack of frontal sinus pneumatization (49%) and lack of mastoid pneumatization (81%).

The hypocalcification defects of the enamel were described by Seow (1991) as normal-thickness enamel, which consists of poorly mineralized matrix, resulting in early loss of the surface and a yellow-brown appearance. Radiographically, enamel is less opaque than dentin and may have a moth-eaten appearance [30]. 

Radiographs and sections of teeth show taurodontic defects in anterior as well as posterior teeth with high pulp horns reaching the dentino-enamel junction [4, 22]. Taurodontism is defined as a developmental condition in which the body of a tooth is elongated at the expense of the root, leading to a large pulp chamber. The pulp chamber is not only large, but it is enlarged at the expense of the body of the tooth. The bifurcations or trifurcations of molar teeth are apically placed, and the external outline of the tooth has a block configuration [12].

Taurodontism is characterized by a large pulp chamber and an altered external outline form of the tooth to distinguish it from other conditions with enlarged pulp chambers that retain the usual shape of the teeth (cynodontism) such as in vitamin-D-dependent rickets, vitamin-D-resistant rickets, pseudo-hyperparathyroidism, and hypophosphatasia [12].

Taurodontism is observed in approximately 3–8% of the general population [31–33]. But it may be an accompanying sign in dental hypodontia and ectodermal dysplasia, Down, Klinefelter, and other dysmorphic syndromes. The mandibular permanent second molar is the tooth most frequently involved [34]. Therefore, presence of taurodontism in the permanent mandibular first molar in TDO is characteristic.

A very recent study compared the craniofacial variations between 53 TDO affected and 34 unaffected family members through cephalometric measurements. This study contained the largest sample of TDO affected and unaffected family members evaluated to date, which aimed to characterize the craniofacial features of TDO syndrome and reveal the role of DLX3 in human craniofacial development. Marked variability was found in craniofacial measurements in both groups. However, TDO affected subjects showed smaller SNB angle, ANB angle, longer mandibular corpus length, and shorter ramus height. Affected individuals varied from severe Class III appearances to moderate Class II with mandibular retrusion. The study showed no difference in mandibular protrusion (SNB angle) between TDO affected and unaffected individuals. Yet, due to a retrusive maxilla, the position of the mandible to the maxilla (ANB angle) appears relatively more prognathic with a Class III skeletal appearance. The study found a statistically significant association between the DLX3 gene mutation in TDO syndrome and increase in mandibular body length, and decrease in ramus height. It is unknown whether this Class III skeletal relation observed in TDO individuals is due to familial factors or a mutation in the DLX3 gene, and further studies are needed to evaluate this [29].

## 5. Diagnosis of TDO

Diagnosis of TDO is not always straightforward. Seow [12, 26] proposed that a precise identification of TDO should include the following major criteria: (1) generalized enamel defects (assessed by clinical and radiographic examination) that show hypomaturation or hypocalcification occurring with enamel hypoplasia; (2) severe taurodontism of the teeth, involving the mandibular first permanent molars (assessed by radiographic examination); (3) an autosomal dominant mode of inheritance; (4) at least one of the other minor features [i.e., nail defects (e.g., brittle and peeling), bone sclerosis (as assessed by lateral cephalograms), and curly, kinky, or wavy hair present at a young age (verified in infancy pictures), that may straighten out later].

Examination of family members shows a high penetrance of the defect, affected members may have missing teeth or may even be edentulous compared with unaffected members of the family who usually have a normal dentition; this may result from extraction of affected teeth due to dental abscesses. Furthermore, genetic testing of suspected family members shows a mutation in the DLX3 gene [13].

The mandibular first permanent molar is considered as a key tooth for assessment of taurodontism due to the fact that any alteration in this tooth, which is considered as the most stable in its series, would indicate a true morphological aberration of the molars. Moreover, the outline of this tooth is clear on panoramic radiographs in contrast to the maxillary molars [31]. A comparison to normal control patients showed that the maxillary second permanent molar was found to have the highest prevalence of taurodontism of a mild type [26].

Therefore, since severe taurodontism is only manifested in TDO, Seow [26] proposed that the severity of taurodontism in addition to the involvement of the mandibular first permanent molars may be used to accurately diagnose TDO syndrome. The severity of taurodontism in the mandibular first permanent molar may be assessed radiographically as proposed by Seow and Lai [31] by measuring the crown-body : root ratio (cb : r) to differentiate different degrees of taurodontism. The cb of each molar can be obtained from the OPG by measuring the length along the vertical axis of the tooth from a perpendicular line drawn through the occlusal pit to a perpendicular line drawn through the furcation. The root (r) length can be determined along the same axis from the furcation to the root apices. From the cb : r ratio, teeth can be classified into 4 types. These are shown in Table 2.

## 6. Differentiation of TDO from Amelogenesis Imperfecta (AI)

Pindborg [35] described TDO as a syndrome manifesting amelogenesis imperfecta (AI), taurodontism, curly hair, and sclerotic bones. He mentioned that the teeth become abscessed within the first years of life because they exhibit very thin, pitted, yellowish-brown hypoplastic and/or hypocalcified enamel. The molars both primary and permanent were described as taurodont in form.

Confusion often exists in clinical diagnosis of AI and TDO because the enamel hypoplastic defects in TDO are similar to those of Witkop's AI Type-IV hypomaturation-hypoplastic variant [36]. Also, uncertainty in diagnosis may result from the clinical variability in the expression of defects in TDO because affected patients may not show the full triad of TDO features [7, 8] especially, with regards to hair, nail, and bone defects which may be mildly expressed [12]. Furthermore, the confusion is due to the reason that taurodontism has been reported as a diagnostic feature of both conditions, and somehow, subjective criteria have been used to determine taurodontism in TDO and AI without comparison to healthy control subjects [26].

The AI variant included in the Witkop [36] classification of AI as Type IV hypomaturation-hypoplastic with taurodontism and further subdivided into type IVA (hypomaturation-hypoplastic, autosomal dominant) and type IVB (hypoplastic-hypomaturation, autosomal dominant) should actually be eliminated as a distinct entity of AI and must be excluded when diagnosing AI as stated by Seow [26]. This is because taurodontism is not a constant feature of any of the types of AI including type IV, especially that the prevalence and severity of taurodontism in primary AI and particularly the AI-Type IV variant was not found to be significantly different from those seen in healthy control subjects.

Hence, the use of taurodontism of the mandibular first permanent molar as a key feature in TDO is important for diagnosis and differentiation of TDO from other defects. The consistent feature of taurodontism in TDO indicates that in this syndrome a defect in ectodermal cells not only results in abnormal amelogenesis, but also in defective root formation [26]. This hypothesis is supported by observations that taurodontism occurs in patients with other types of ectodermal aberrations as ectodermal dysplasia [37] as well as hypodontia [31]. Taurodontism is associated with several conditions including chromosomal anomalies such as Klinefelter syndrome [38] and trisomy 21/Down syndrome [39]. It may also be associated with Mohr syndrome (Oral-facial-digital syndrome II), [40] microcephalic dwarfism, [41] and ectodermal dysplasia [42]. Taurodontism unassociated with a syndrome is most likely a polygenic trait [43–45].

The association between TDO and AI-Type IV variant was further evaluated by Price et al. [46] through mutational analysis and sequencing studies. They found that neither the affected nor unaffected subjects possessed the 4 bp DLX3 mutation present in TDO affected individuals. The authors concluded that TDO and AI-Type IV were genetically distinct conditions.

Dong et al. [47] studied subjects affected with AI-Type IV autosomal dominant variant who showed reduced enamel thickness, enlarged pulps, and no curly hair or evidence of bone sclerosis on skull radiographs. The authors excluded the diagnosis of TDO due to a lack of bone and hair involvement. They were able to map an affected family to human chromosome 17 q21-q22 and identified a novel 2 bp deletion DLX3 mutation located within the homeodomain of DLX3. This was the first report of a mutation within the homeodomain of DLX3 compared to previous studies that have shown a DLX3 mutation outside the homeodomain associated with TDO; the authors concluded that TDO and some forms of AI-Type IV variant are allelic and suggested that this new DLX3 mutation affects the formation of enamel and teeth but lacks the defects of bone and hair.

Later, Wright et al. [48] identified another group of TDO kindred in a family in Switzerland with the same 2 bp deletion mutation. All affected subjects had hair defects and reduced enamel thickness; however, tooth size and severity of taurodontism were less severe compared to the 4 bp deletion mutation. The family did not exhibit sclerotic bone on skull radiographs. The authors concluded that this new 2 bp deletion caused an attenuated phenotype of TDO syndrome with less severe hair, tooth, and bone manifestations and is not an AI-Type IV variant.

In order to uncover genetic etiology, a mutational analysis study on a Korean family with overlapping phenotypes of TDO and AI-Type IV was performed. The identified mutation was a 2 bp deletion mutation in the DLX3 gene. The subjects displayed hypomature and hypoplastic enamel, with no characteristic taurodontic features, or increased bone density. However, the affected individuals had brittle nails and curly hair at birth. The study concluded that the 2 bp deletion mutation had not only enamel defects, but also other clinical phenotypes resembling those of an attenuated phenotype of TDO syndrome [49].

## 7. Management Strategies

A team approach is required in management of TDO; members of the dental team may include the paediatric dentist, orthodontist, endodontist, oral surgeon, prosthodontist, and oral radiologist. Other members include the pediatrician, medical radiologist, and geneticist. Proper documentation of a case includes radiographs (intra-oral, orthopantograms and lateral cephalograms), study casts, and good quality extraoral and intraoral photographs. After a problem list is formulated, a preliminary short-term treatment plan is established upon diagnosis, as well as a detailed long-term plan for followup in the coming years.

The main clinical problems experienced by the patients with TDO are hypersensitivity of the teeth, loss of occlusal vertical dimension due to attrition and loss of tooth structure, dental abscesses resulting from pulp exposures, esthetics, and psychosocial problems. Additional problems may include skeletal- and orthodontic-related problems. The main aim in managing affected patients focuses on preventing clinical problems as early as possible such as attrition, caries and pulpal infection, relieving pain associated with sensitive teeth and dental abscesses, restoring function, maintaining the occlusion and occlusal vertical dimension, and improving dental esthetics [12].

Preventive therapy is essential in order to reduce sensitivity and dental caries. Two types of therapeutic products can be considered for sensitivity in terms of mode of application: professional and self-applied products. In patients with mild to moderate sensitivity, a noninvasive approach to management is indicated including diet counselling, oral hygiene instructions, and use of self-applied products at home. Diet advice includes reducing acidic foods, intake of alkaline (milk), or at least neutral (water) after acidic drinks, and use of a straw to sip the drink and avoid swishing it around the teeth [50]. Oral hygiene instructions should be given to the patient to use a toothbrush with soft bristles and desensitizing toothpastes; the patient should be advised to use a minimal amount of water to prevent dilution of the active agent. In addition, prescription of mouth rinses and chewing gums containing potassium nitrate or sodium fluoride is indicated [51].

Recently, the casein milk protein has been used to develop a remineralizing agent (GC Tooth Mousse). The casein phosphopeptide (CPP) contains phosphoseryl sequences, which are attached and stabilized with amorphous calcium phosphate (ACP). The stabilized CPP-ACP prevents the dissolution of calcium and phosphate ions and maintains a supersaturated solution of bioavailable calcium and phosphates [52]. Several studies have shown that CPP-ACP can effectively remineralize enamel subsurface lesions [53, 54]. Due to its remineralizing capacity, it has also been proposed by the manufacturers to help in prevention and treatment of dentin sensitivity. Another recent advance is a toothpaste containing 8.0% arginine and calcium carbonate, known as Pro-Argin technology. There is clinical evidence for the superior efficacy of this toothpaste versus a potassium-based desensitizing toothpaste and delivery of superior instant and lasting relief of hypersensitivity compared to a toothpaste containing 8% strontium acetate [55–57].

In cases of severe intensity, a high-concentration fluoridated varnish in office treatment can offer good results. If such management measures prove ineffective, semi-invasive therapy can be decided, particularly if the disorder affects the life style of the patient. This includes use of agents that can polymerize (dentinal adhesives) or set (glass ionomer and composite resin) within the dentinal tubules, thereby occluding the latter [51]. 

Bioglass has been reported to promote infiltration and remineralization of dentinal tubules. The basic component is silica, which acts as a nucleation site for precipitation of calcium and phosphate. SEM analysis has shown that bioglass application forms an apatite layer, which occludes the dentinal tubules [58]. Laser irradiation (Nd-YAG, GaAlAs, and Er-YAG lasers) may also be used for desensitizing sensitive teeth; the mechanism is not fully understood, but may be through occluding dentinal tubules, blocking the movement of fluid inside the dentinal tubules by coagulation of proteins, [59] and affecting the neural transmission in the dentinal tubules [60]. A recent progress in the treatment of hypersensitivity is by use of calcium silicate cement derived from Portland cement, which helps to occlude the dentinal tubules by remineralisation [61].

Extreme cases of sensitivity characterized by material or substance loss require invasive treatment such as placement of crowns, endodontic treatment, or tooth extraction [50]. Sensitivity of the teeth and attrition in young patients—leading to pulp exposures, abscesses, endodontic treatment or extractions of teeth, and loss of occlusal vertical dimension—may be treated by stainless steel crowns as soon as the molars have reached proper occlusal height [62]. The advantage of these crowns is durability; they offer full surface coverage, require no further intervention other than regular followup, cause no insult to the pulp, and are an interim measure that can be replaced later with porcelain jacket crowns [63, 64].

In order to conserve tooth structure, crown placement on molars may involve a technique, where no crown preparation is required [12, 62]. In this technique glass ionomer cement, resin modified glass ionomer, or composite resin is placed on the occlusal surface of a partially erupted molar so that the tooth erupts with the resin in occlusion with the opposing teeth; this will minimise any attrition of occlusal tooth structure. In the preparation of the stainless steel crowns, occlusal reduction is then performed on the restorative materials [12]. Proximal reduction is minimised by the prior placement of separating elastics through the contact points prior to crown placement [65]. Upon cementation of the crown, glass ionomer cements are the material of choice [12].

Prior to crown placement, restoration of carious and hypoplastic defects in teeth is achieved with adhesive materials such as glass ionomer cements or resin modified glass ionomer placed on nonhypomineralized enamel since the weak enamel margins tend to fracture away. Glass ionomer offers the advantage of adhesion to tooth structure by a chemical bond to dentine, insulation, easy handling characteristics and no technique sensitivity, and fluoride release [66]. Dental amalgam is of limited value for hypomineralized teeth because it is nonadhesive, prone to marginal leakage, offers no mechanical support of the tooth, and is a poor insulator [67].

The discoloration of the anterior teeth may require full-coverage porcelain jacket crowns. However, this is not recommended in the young child who is less than 16 years of age due to large pulps prone to exposure during crown preparation in addition to premature gingival contours [68]. Temporary veneer restorations using direct composite resin facings or porcelain veneers (without or with very minimal preparation) may be considered [12, 68].

Endodontic management in molars may be required in certain cases. Peretz et al. [69] reported only 36% success rate for RCT of permanent first molars in children aged 8–16 years old, because of underdeveloped dentinal walls, lack of apex formation, and wide canals. This can be minimized by novel techniques such the use of MTA as an apical plug in immature teeth followed shortly by an intracanal filling material. It seems that the taurodont form does not interfere with routine operative procedures, but it is suggested that the morphology might hamper the location of orifices and could create difficulties in instrumentation and obturation in endodontic treatment [44]. Endodontic treatment of a taurodontic tooth is challenging, because it requires special care in handling and identifying the number of root canals [70]. Successful endodontic treatment of taurodontism has rarely been reported [71].

Extraction of poor prognosis, unrestorable, severely hypoplastic first permanent molars might be the last treatment option in some cases. The optimal time for this is at the beginning of calcification of the bifurcation of roots of the second permanent molar, usually around 8.5–9.5 months. In practice, this will mean extraction of the mandibular molars followed 6 months later by extraction of the maxillary molars. Orthodontic and pediatric opinions should be sought before the extractions are done [72].

## 8. Management Problems Related to Dental Treatment

Management problems that may arise include behaviour management related to dealing with young patients due to fear and anxiety. To minimize this, local anesthesia should be used to decrease sensitivity associated during the procedure; nitrous oxide sedation may also be useful with local anesthesia in apprehensive patients, and in some cases treatment under general anesthesia may be required. Access and isolation posteriorly may be another management problem, the use of a rubber dam will enhance access, isolation, and comfort, and furthermore, it helps isolate other sensitive teeth avoiding their exposure to cold air or water [67, 72].

Other management problems are those associated with clinical treatment of taurodontic teeth due to abnormal morphology. As mentioned above, endodontic challenges are related to a pulp chamber with increased length hindering location, cleaning and obturation of root canals. Besides, the frequent presence of pulp stones and unusual apical root canal systems may further complicate treatment [71, 73]. A modified filling technique, consisting of combined lateral condensation in the apical region with vertical condensation in the extended pulp chamber, has been described and recommended [44, 74]. It has been proposed that vital pulpotomy may be considered as the treatment of choice in cases of hypertaurodontism (pulp chamber nearly reaches the apex and then breaks up into two or four channels) instead of routine pulpectomy [75].

Challenges faced with prosthodontics and orthodontics are related to the fact that taurodontic molars should not be considered as adequate abutment teeth due to decreased surface area embedded in the alveolus and, therefore, they may not have sufficient resistance to lateral displacing forces as compared to cynodont teeth [44]. Also, the absence of a cervical constriction decreases the buttressing effect against excessive loading of the crown [76]. In addition, the placement of posts is not recommended and should be avoided for prosthetic tooth reconstruction [70].

For the same reason related to little surface area embedded in the alveolus, extraction of taurodontic molars should be easier compared to cynodont teeth as long as the roots are not widely divergent [44]; however, complications have been reported because of a dilated apical third [71]. It is believed that the difficulty in extraction of hypertaurodontic teeth is due to the increased apical shift of the furcation creating a difficulty in gripping the tooth firmly with forceps beaks thus, use of surgical elevators is highly indicated in such cases [76].

From a periodontal aspect, taurodontism may be considered as advantageous due to the apical location of the furcation area consequently making it less susceptible to periodontal disease. When periodontal pocketing or gingival recession occurs, chances of furcation involvement are much less compared to those in cynodont teeth because taurodont teeth significant periodontal destruction must take place before furcation involvement occurs [75].

In conclusion, TDO is a syndrome with important implications for dentists. The challenges faced with regards to this syndrome are related to establishing an accurate diagnosis and providing the patient with a life-long prevention protocol and delivering proper treatment strategies according to the age and needs of the patient that aim to decrease the symptoms, maintain the dentition, and when implemented early enough, result in a long-term excellent prognosis.

## Figures and Tables

**Table 1:** Characteristic defects in TDO.

Defect	Reported expression of defect/features	Reference
(1) Hair defects	Kinky or tightly curled hair at birth	[1–3, 16–19]
	Wavy hair	[7, 18]
	Curly hair at birth that straightened out a few years later	[6]

(2) Dental defects	Yellow-brown discolored teeth	[1–6, 9, 16–18]
	Thin enamel associated with hypocalcification or hypomaturation defects and enamel hypoplasia	[1–8, 12, 16–19]
	Severe attrition of enamel	[1, 2, 5, 8, 16, 17, 19]
	Dental abscesses	[1–4]
	Taurodontism in both the primary and permanent dentitions	[13]
	Taurodontism in the first permanent molar (key tooth)	[20]
	Histologic sections show:	
	(i) hypocalcified enamel that is decreased in thickness with enlarged pulp chambers	[2, 3, 7]
	(ii) small amounts of interglobular dentin have been noted in a few teeth	[6]

(3) Bone changes	Sclerosis may be a variable feature. It is commonly reported in the following areas: base of the skull, mastoids and zones of provisional calcification in the long bones	[1–3, 22]

(4) Nail defects	Splitting of the superficial layers of the nails	[1–3, 7, 8]
	Sometimes, only some toenails may be affected	[21]

(5) Craniofacial defects	Frontal bossing	[1, 7]
	Square jaw	[1]
	Mandibular prognathism	[2]
	Maxillary retrusion	[29]
	Dolichocephaly	[1, 9]

(6) Other reported abnormalities	Impacted teeth	[4, 7]
	Clinodactyly	[1]
	Skin lesions	[2]

**Table 2:** Crown body: root ratio classification according to Seow and Lai [31] to differentiate different degrees of taurodontism.

Degree of taurodontism	Crown body : root ratio (cb : r ratio)
(1) Cynodont (normal)	<1.10
(2) Hypotaurodontic	1.10–1.29
(3) Mesotaurodontic	1.30–2.00
(4) Hypertaurodontic	>2.00
